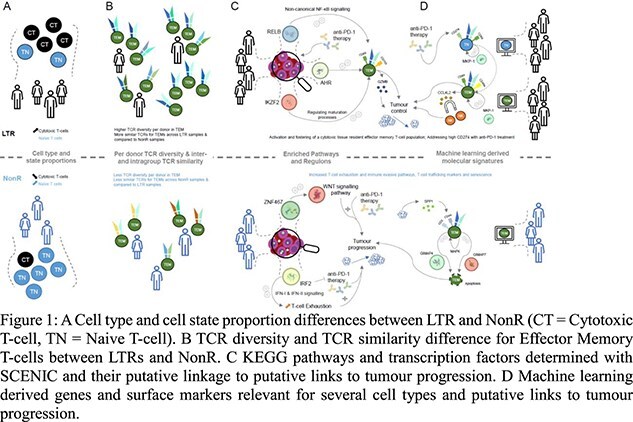# Blood memory CD8 T cell phenotypes in lung cancer patients predict immune checkpoint treatment responses

**DOI:** 10.1093/bib/bbaf631.001

**Published:** 2025-12-12

**Authors:** Florian Schmidt, Kan Xing Wu, Yovita Ida Purwanti, Nicholas Tan, Daniel Carbajo, Bok Ke Xin, Andreas Wilm, Michael Fehlings, Daniel MacLeod, Alessandra Nardin, Daniel Tan, Katja Fink

**Affiliations:** ImmunoScape Pte. Ltd., Singapore; ImmunoScape Pte. Ltd., Singapore; ImmunoScape Pte. Ltd., Singapore; ImmunoScape Pte. Ltd., Singapore; ImmunoScape Pte. Ltd., Singapore; National Cancer Centre Singapore, Singapore; ImmunoScape Pte. Ltd., Singapore; ImmunoScape Pte. Ltd., Singapore; ImmunoScape Pte. Ltd., Singapore; ImmunoScape Pte. Ltd., Singapore; National Cancer Centre Singapore, Singapore; ImmunoScape Pte. Ltd., Singapore

## Abstract

**Motivation:**

T cells are central to the immune response against tumours but are frequently suppressed within the tumour microenvironment through intrinsic inhibitory pathways.

Immune checkpoint inhibitor (ICI) therapy—using monoclonal antibodies targeting inhibitory T cell receptors—has transformed cancer treatment, including for non-small cell lung cancer (NSCLC) [1]. However, some patients are ineligible for ICI therapy due to low expression of inhibitory receptors in tumour biopsies or a low tumour mutational burden (TMB), both of which are associated with reduced response rates [2]. Therefore, improving patient stratification methods remains a critical need.

**Cohort and Methods:**

We analysed blood-derived CD8+ T cells from NSCLC patients, who exhibited prolonged responses to ICI therapy, hypothesizing that CD8+ T cells play a key role in these patients to control their tumours. We used single cell sequencing technology to deeply phenotype (single cell VDJ-CITE-seq) these cells and compared data from long-term responders (LTR) (range: 4–32 months) to new-on-treatment non-responder (NonR) and responder patients, both at baseline and several weeks after the initiation of treatment. After quality control, we obtained 104,400 cells representing data from 10 LTR samples from 9 donors, a single sample from a responder patient and 7 NonR samples from 3 NonR donors.

**Results:**

In this study, we investigated molecular signatures associated with ICI response using deep phenotypic single-cell multi-omics profiles of blood derived CD8+ T-cell samples from NSCLC samples contrasting LTR and NonR samples [3]. While there were no significant differences in cell type distribution between the two patient groups at the conventional 0.05 threshold, we noticed a trend of naive T cell (TN) depletion in LTRs compared to NonR (Wilcoxon, p = 0.088). The same applies comparing cell states; here we found that LTR patients tend to have more cytotoxic cells compared to non-responders (Wilcoxon, p = 0.088) (Fig. 1A). We further examined the TCR repertoire in LTR (n = 10) and NonR (n = 7) samples across all time points. We observed that the clone sizes of unique paired TCRs increased with maturity of the T cell subtypes and that the LTR samples have a more diverse set (Simpson Index) of clonotypes (i.e., more unique TCRs) within the cytotoxic TEM and TEMRA populations compared to NonR samples. In addition, we investigated the across group TCR similarity by comparing the mean *TCRDist* similarity across all possible comparisons between samples. We hypothesized that if the ICI response is indeed driven by tumour specific T-cell, the TCR space of LTRs should be more similar within the LTR group itself than towards NonR as well as NonR compared among each other. We found that this is indeed the case (Fig. 1B). We next sought to identify biomarker signatures that could potentially be useful to predict clinical responses. We used statistical tests to determine whether any potentially confounding covariates from the recorded metadata should be included in a machine learning model. We found neither gender (chi^2^ test, p = 0.21), tumour proportion score (TPS) (t-test, p = 0.87), nor age (t-test, p = 0.76) are associated to response. For, the association between treatment regimen at response and response itself, a chi^2^ test did show a significant association (p = 0.004). However, we decided not to include the treatment regimen at response as a feature to allow for both the incorporation and applicability of the model to new on treatment baseline samples. Given the relative stability of T cell subset and state distribution in longitudinal samples, we included all patient visits for the training of machine-learning models and used lenient cut-offs on pseudo bulk derived differentially expressed genes and surface markers to construct a candidate feature matrix as input for our machine-learning models in a T cell subset specific manner. Next, we applied data balancing to construct training and test data sets in a leave-one-sample-out cross validation procedure to assess model performance both on single cell, and sample level, whereby we classify a sample to be correctly predicted if more than 50% of single cells have been assigned the correct label. Multiple time points per patient are considered as separate samples in a per-sample evaluation. In 93% of NKT-like and 91% of TSCM, CM and TN single cells were correctly assigned to belong to a non-responder. The best results on sample level were observed for the TEM model that predicted all ten responder samples correctly. In a leave-one sample out cross validation, including a lung responder sample, the TEM model predicted 10 out of 10 LTR, 1 out of 1 R and 6 out of 7 NonR samples correctly. Compared to a clinical standard metric, the tumour proportion score (TPS), our TEM-specific model was able to correctly identify, a non-responder that was otherwise TPS high and five LTRs that were TPS low. Importantly, the analysed dataset included one baseline sample from responder sample, which was predicted correctly as responder even though the model was trained with mostly on-treatment samples. A literature search of all features with a non-zero regression coefficient in the TEM model revealed that for 22 of 23 features, prior studies suggest an involvement of the respective genes or surface markers in the T cell immune response or lung cancer biology, respectively. Combined with a regulon analysis (*SCENIC*) as well as a Gene Set Enrichment Analysis, the features highlighted by the model gave rise to several hypothesis related to the LTR and NonR phenotype (Fig. 1C-D). Despite limited sample sizes and challenging data collection, our signature aligned with findings from two external scRNA-seq datasets. Using module scores, we demonstrated that our signature separates NSCLC patients in two independent cohorts: (1) 92.000 single cells from 3 pre-treatment and 12 post-treatment patients grouped by major pathologic response [4] and (2) 222.144 cells from 33 patients grouped by ICI efficacy and immune-related adverse events [5]. Through comprehensive molecular profiling of NSCLC patients, we identified factors correlated with ICI response. The integrative nature of our machine learning approach is well-suited in providing a more nuanced and comprehensive look at the complexities of ICI therapeutic response that otherwise traditional approaches, such as TMB and single biomarker detection, are unable to fully capture. Importantly, our findings are derived from circulating T cells—a minimally invasive source—highlighting the potential for improved screening methods to identify patients likely to benefit from ICI therapy.

**References:**

1. Arafat Hossain, M. A comprehensive review of immune checkpoint inhibitors for cancer treatment. Int. Immunopharmacol. 2024, 143(Pt 2):113365.

2. Marabelle, A., et al. Association of tumour mutational burden with outcomes in patients with advanced solid tumours treated with pembrolizumab: prospective biomarker analysis of the multicohort, open-label, phase 2 KEYNOTE-158 study. Lancet Oncol 2020 21(10):1353–1365.

3. Schmidt, F., et al. Blood memory CD8 T cell phenotypes in lung cancer patients predict immune checkpoint treatment responses. Front Oncol. 2025, 15:1629802.

4. Hu, J., et al. Tumor microenvironment remodeling after neoadjuvant immunotherapy in non-small cell lung cancer revealed by single-cell rna sequencing. Genome Medicine 2023, 15(1):14.

5. Kim, G. D., et al. Single-cell RNA sequencing of baseline PBMCs predicts ICI efficacy and irAE severity in patients with NSCLC. J. Immunother. 2025, 13(5):e011636.